# Institutions for Sailors

**Published:** 1915-01-02

**Authors:** G. King Hall

**Affiliations:** Director, Late Commander-in-Chief of the Australian Squadron.


*To the Editor of* The Hospital.

### Institutions for Sailors.

*To the Editor of* The Hospital.

Sir,—Winter is upon us, and as a sailor may I claim your sympathetic attention?

We have felt it a privilege since war began to co-operate with the authorities in supplying national needs; raising money for the National Relief Fund; opening our Sailors' Institutes as Red Cross hospitals, refugees' homes, recreation and concert rooms, canteens and Gospel halls for the young men of our new Army; giving a Testament, a handshake, and a word of inspiration to thousands of sailors and soldiers leaving our shores; providing books and magazines for the sailors afloat and the soldiers using our branches; finding more than twenty thousand warm garments and woollens for the blue-jackets in the North Sea, for military hospitals on both sides of the Channel, and for all our lighthouse and lightship keepers round the United Kingdom.

Much of our chaplains' work in and near the war zone—at Hamburg, Rotterdam, Antwerp, Havre, St. Nazaire, etc.—may not and could not be portrayed in words, but the nation and our brave defenders would be the poorer, body and soul, were it not for the unselfish devotion of our port missionaries and their staffs.

We are compelled to tell our friends that now, after doing our duty in undertaking this national work, with its largely increased responsibilities, £10,000 at least is urgently needed to repay bankers' overdrafts and to save our workers from undue financial anxiety.

Our Treasurer, Sir Frederick Green, J.P. (who has recently sent us a cheque for £500), will gladly receive any gifts towards the above, or they may be sent to me at the above address. Pennies and pounds will be helpful and most welcome. The British and Foreign Sailors' Society recognises the good providence of God and asks for a thank offering.—Very faithfully yours,

G. King Hall, Director,
Late Commander-in-Chief of the
Australian Squadron.

